# 

**Published:** 1880-09

**Authors:** 


*CONTENTS.*

| | Page | | Page |
|---|---|---|---|
| Pure Food | 141 | Drinking and Death | 154 |
| Weakly Youths | 142 | Liquor Drinking | 155 |
| Walk Softly | 142 | Aristocracy | 156 |
| Beautiful Old Age | 143 | Early Marriages | 157 |
| Living Ages | 144 | Fruits in Summer | 158 |
| Object of Eating | 145 | Care of the Eyes | 159 |
| Leaden Water-Pipes | 146 | Damp Walls | 162 |
| Cheapest Food | 147 | Safety of Families | 163 |
| The Grave of Hope | 147 | Laughter and Music | 165 |
| Gratuitous Medical Advice | 150 | The True Physician | 166 |
| Children Eating | 150 | Avenues of Death | 166 |
| Temperament Differences | 151 | Cure of Corns | 168 |
| Well Done | 153 | Smoking Tobacco | 168 |
| Baths and Bathing | 153 | Preventing Disease | 170 |
| Quenching Thirst | 154 | Greed of Gold | 170 |

